# Complete Genome Sequences of Erwinia amylovora Phages vB_EamP-S2 and vB_EamM-Bue1

**DOI:** 10.1128/MRA.00891-18

**Published:** 2018-07-26

**Authors:** Leandra E. Knecht, Yannick Born, Joël F. Pothier, Martin J. Loessner, Lars Fieseler

**Affiliations:** a Food Microbiology Research Group, Institute of Food and Beverage Innovation, Zurich University of Applied Sciences (ZHAW), Wädenswil, Switzerland; b Institute of Food, Nutrition and Health, ETH Zurich, Zurich, Switzerland; c Environmental Genomics and Systems Biology Research Group, Institute of Natural Resource Sciences, Zurich University of Applied Sciences (ZHAW), Wädenswil, Switzerland; University of California, Riverside

## Abstract

Phages vB_EamP-S2 (S2) and vB_EamM-Bue1 (Bue1) infect the plant pathogen Erwinia amylovora. S2 has a genome size of 45,495 bp and belongs to the genus SP6virus. The genome size of Bue1, related to Salmonella phage Vil, is 164,037 bp. Both phages possess a depolymerase enzyme, a frequent feature of E. amylovora phages.

## Abstract (precis)

Phages vB_EamP-S2 (S2) and vB_EamM-Bue1 (Bue1) infect the plant pathogen Erwinia amylovora. S2 has a genome size of 45,495 bp and belongs to the genus SP6virus.

## ANNOUNCEMENT

The enterobacterium Erwinia amylovora is the causative agent of fire blight, a plant disease affecting pome fruit (1). The antibiotic streptomycin is widely used to control the disease (2). However, potential resistance development and public demand for environment-friendly alternatives promote the development of new control strategies (3). One alternative is bacteriophage treatment. E. amylovora-specific phages vB_EamP-S2 (S2) and vB_EamM-Bue1 (Bue1) were isolated from soil samples (Swiss apple orchards). Both phages possess a broad host range, infecting 83% (S2) and 96% (Bue1) of the E. amylovora strains tested. Transmission electron microscopy identified S2 as a podovirus (4), with an average capsid size of 64 nm (±4.6 nm), and Bue1 as a myovirus, with an average capsid size of 79 nm (±2.4 nm) and a 126-nm-long (±7.4 nm) contractile tail. Phage DNA was extracted as described previously (4) and sheared into 550-bp fragments on an E220 ultrasonicator (Covaris, Woburn, MA). Libraries were prepared on a NeoPrep system (Illumina, San Diego, CA) using a TruSeq Nano DNA kit (Illumina) with six PCR cycles, according to the manufacturer’s instructions. Paired-end sequencing of 300 bp was performed on a MiSeq instrument (Illumina) using a 600-cycle MiSeq reagent kit version 3 (Illumina), according to the manufacturer’s instructions. This generated 4,387,300 (S2) and 4,642,900 (Bue1) raw reads. *De novo* assemblies were created using SeqMan NGen (Lasergene Genomics package version 12.1.0; DNAStar, Madison, WI). The average coverages were 5,463× (S2) and 7,668× (Bue1).

Coding sequences (CDS) were annotated using RAST 2.0 (5) and BLAST (6) comparisons with the nonredundant GenBank database. ARAGORN (7) and tRNAscan-SE 2.0 (8) identified tRNA sequences. Overall nucleotide sequence identities were analyzed using EMBOSS stretcher (9).

The S2 genome is 45,495 bp long. Primer walking toward the expected ends determined direct terminal repeats (297 bp). The G+C content is 49.8%. Of the 49 CDS annotated, 26 were assigned a putative function. No tRNA was found. S2 shares a nucleotide identity of 76.7% with E. amylovora phage Era103 (GenBank accession number NC_009014; SP6-like) and 54.1% with Salmonella phage SP6 (GenBank accession number NC_004831), the type species of the genus SP6virus (10), placing S2 into the subfamily Autographivirinae, genus SP6virus.

The double-stranded linear DNA of Bue1 is 164,037 bp long, with a G+C content of 50.2% containing 175 annotated CDS, with 64 with assigned putative functions and one tRNA^Lys^ sequence. The circularly permuted/terminally redundant genome was opened upstream of the rIIA lysis gene for annotation. Due to the nucleotide identity of 92.1% with E. amylovora phage phiEa2809 (GenBank accession number NC_027340) and 52.9% with the Salmonella phage Vi01 (GenBank accession number NC_015296), Bue1 can be assigned to the family Ackermannviridae (11).

Both S2 and Bue1 encode putative exopolysaccharide (EPS) depolymerases, which degrade the amylovoran component of the host’s capsule (12). Similar genes are present in E. amylovora phages vB_EamP-L1 (GenBank accession number NC_019510; T7virus) (4), Ea9-2 (GenBank accession number NC_023579; Ea92virus) (13), and phiEa2809 (14). This widespread prevalence of EPS depolymerases among E. amylovora podoviruses and myoviruses indicates an importance in host infection and specificity.

### Data availability.

The annotated sequences of the two Erwinia amylovora phage genomes were deposited at GenBank under the accession numbers MG736918 (vB_EamP-S2) and MG973030 (vB_EamM-Bue1).
